# An overview on current molecular tools for heterologous gene expression in *Trichoderma*

**DOI:** 10.1186/s40694-021-00119-2

**Published:** 2021-10-26

**Authors:** Irene Tomico-Cuenca, Robert L. Mach, Astrid R. Mach-Aigner, Christian Derntl

**Affiliations:** Institute of Chemical, Environmental and Bioscience Engineering, TU Wien, Gumpendorfer Strasse 1a, 1060 Wien, Austria

**Keywords:** Trichoderma, Heterologous protein expression, Biocontrol, Industrial enzymes, Promoters, Genome editing, Protein secretion

## Abstract

Fungi of the genus *Trichoderma* are routinely used as biocontrol agents and for the production of industrial enzymes. *Trichoderma* spp. are interesting hosts for heterologous gene expression because their saprotrophic and mycoparasitic lifestyles enable them to thrive on a large number of nutrient sources and some members of this genus are generally recognized as safe (GRAS status). In this review, we summarize and discuss several aspects involved in heterologous gene expression in *Trichoderma*, including transformation methods, genome editing strategies, native and synthetic expression systems and implications of protein secretion. This review focuses on the industrial workhorse *Trichoderma reesei* because this fungus is the best-studied member of this genus for protein expression and secretion. However, the discussed strategies and tools can be expected to be transferable to other *Trichoderma* species.

## Introduction

**Fig. 1:**
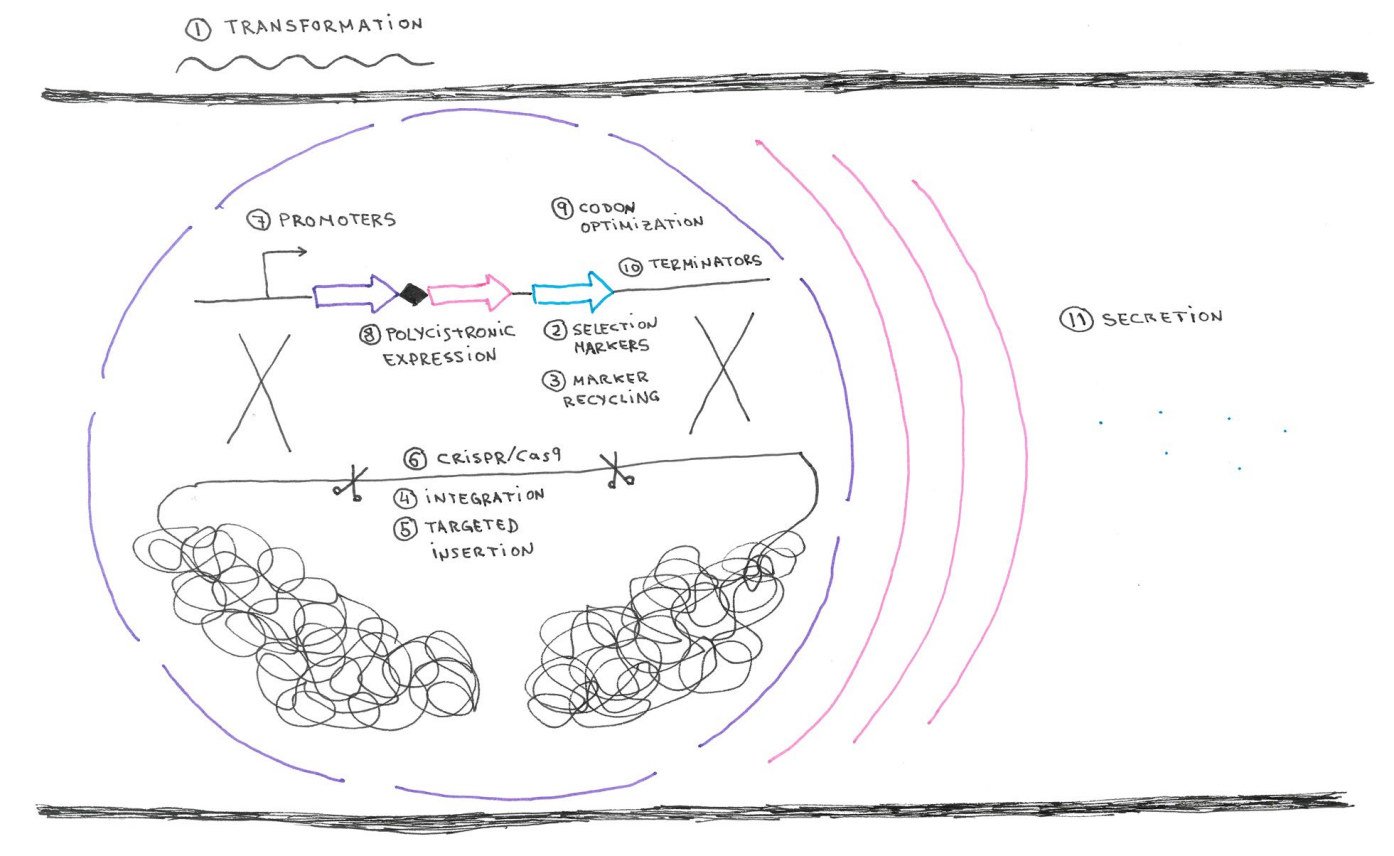
Schematic overview of the aspects to be considered for heterologous gene expression. First, the DNA containing the expression cassette needs to be transformed (1) into the fungal cell. Suitable (ideally reusable) markers (2 + 3) are used to select for ectopic integration or targeted insertion (4 + 5), which might be supported by the CRISRP/Cas9 system (6). For optimal expression, the choice of promoters (7), potentially 2A peptides for polycistronic expression (8), codon-optimization (9), and terminators (10) need to be considered. If a protein is to be secreted (11) a signal peptide needs to be added

The genus *Trichoderma* contains a large number of fungi with mainly saprotrophic and mycoparasitic lifestyles [1]. Based on these properties, *Trichoderma* species are used for the industrial production of enzymes and as biocontrol agents. *T. reesei* is often called an industrial workhorse due to its outstandingly high enzyme production and secretion rates of up to 100 g/l [2]. Its native cellulases and xylanases are routinely used in the paper and pulp industry, the textile industry, the food and feed industry and are a key component for the production of second-generation biofuels [3]. These high enzyme production capabilities, its fast growth rate, and the classification as a GRAS (generally recognized as safe) organism by the FDA [4] made *T. reesei* an obvious choice for heterologous protein production. In 1982, the first heterologous product, calf chymosin, was heterologously produced in *T. reesei* [5], only two years after the development of a transformation system [6]. In the following years and decades, several recombinant proteins with a broad range of origins (fungal, bacterial, plant, and mammalian) were expressed in *T. reesei,* e.g., [7–11]. Based on its broad application and the ongoing research and development regarding heterologous gene expression, this review focuses mainly on *T. reesei*. However, the used strategies and tools can be expected to be transferable to other *Trichoderma* species. In the present review, a collection of currently used techniques and methods for heterologous expression in *Trichoderma* spp. (e.g. aspects regarding transformation, gene expression, and protein secretion) is presented (Fig. 1). Naturally, these strategies and tools can also be used for the (over)-expression of homologous genes.

## Transformation methods

The availability of an efficient transformation method is vital for heterologous gene expression. There are several described methods for filamentous fungi. The most common ones (according to [12]) are: polyethylene glycol (PEG)-mediated transformation of protoplasts [13], *Agrobacterium tumefaciens*-mediated transformation [14], electroporation [15] and biolistic [16]. All of these methods were applied with different success rates in different *Trichoderma* species.

### PEG-mediated transformation

For the PEG-mediated transformation of protoplast, cocktails of cell wall degrading enzymes (mainly chitinases and beta-glucosidases, e.g., lysing enzymes from *Trichoderma harzianum*, Sigma-Aldrich L1412 or Yatalase™ Enzyme, Takara T017) are used to remove the cell walls of hyphae, leaving so-called protoplasts. These protoplasts are extremely sensitive towards chemical and physical stresses and must be maintained in osmo-stabilizing conditions, i.e., high concentrations of sucrose or sorbitol and a light basic pH value. The protoplasts are then transformed with DNA using polyethylene glycol (PEG) and CaCl_2_. Interestingly, a commonly used enzymes cocktail for the degradation of *Trichoderma* cell walls originates from the mycoparasite *T. harzianum*. This method is the most commonly used in *Trichoderma* due to its simplicity, low time consumption and high yield of transformants (200–800 colonies per μg of DNA in *T. reesei*) [13]. It is a simple method, it does not require expensive equipment, and it has been implemented in several *Trichoderma* species, such as *T. harzanium* [17]*, T. atroviride, T. longibrachiatum* and *T. asperellum* [18].

### Agrobacterium tumefaciens-mediated transformation

**Fig. 2:**
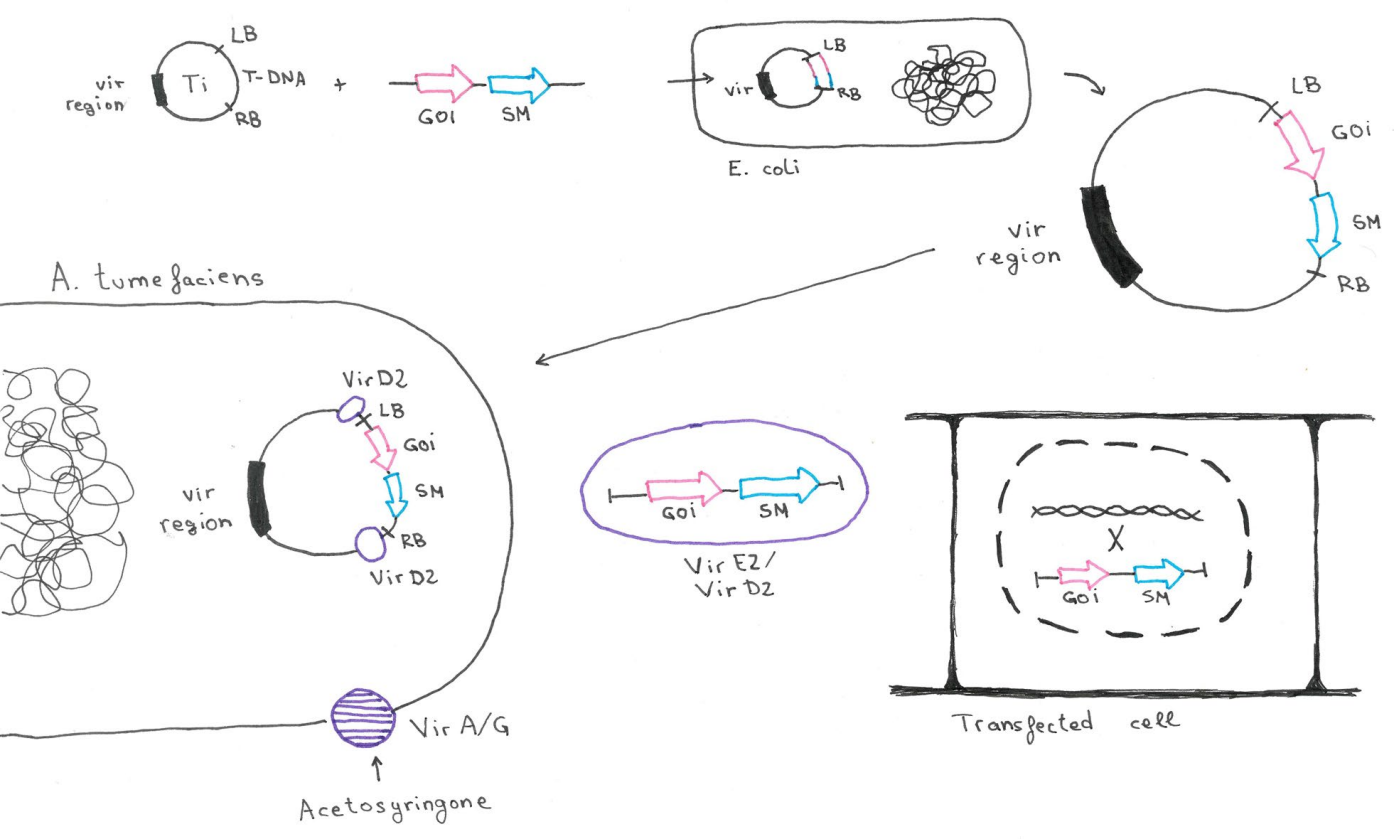
*Agrobacterium tumefaciens*-mediated transformation. The native Ti plasmid contains the *vir* region and the T-DNA that is flanked by left and right borders (LB, RB). The T-DNA can be replaced by a gene of interest (GOI) and a selection marker (SM) by cloning in *E. coli*. The recombinant plasmid is then transformed into *A. tumefaciens*. Upon stimulation with acetosyringone, the VirA/C histidine kinase receptor actives expression of the *vir* genes, which result in release and transformation of the T-DNA (in complex with VirE2 and VirD2) into the host cell and nuclear import

*Agrobacterium tumefaciens*-mediated transformation (ATMT) is based on the native capacity of *A. tumefaciens* to infect plants and integrate part of the Ti plasmid into the plant genome (Fig. 2). The Ti plasmid contains the so-called *vir* region, which comprises approximately 30 genes, organized into six complementation groups: *virA, virB, virC, virD, virE* and *virG*. The expression of the *vir* genes and thus transformation is regulated by the *virA/virG* two-component regulatory system. The histidine kinase VirA reacts to the presence of monosaccharides and phenolic compounds from wounded plants and activates the response regulator VirG, which activates the expression of all other *vir* genes [19]. Notably, the *vir* genes can be activated in vitro by adding acetosyringone, a phenolic inducer [20]. Next, the T-DNA is released from the Ti plasmid [21] with the nickase VirD2 is staying attached to the DNA, and they are co-transformed as a complex into the plant cell. VirD2 contains a nuclear localization signal (NLS). It is thus considered to be necessary for the transport of the T-DNA into the nucleus of the plant cell [22–24]. The T-DNA contains genes for plant growth-stimulating hormones (*aux* and *cyt*) and nopaline biosynthesis. Upon random integration of the T-DNA into the plant genome, the plants form galls and release nopaline, which *A. tumefaciens* can use as a nitrogen source [25].

This natural transformation system can be used for genetic engineering. To this end, usually, a binary vector system is constructed. One plasmid contains the *vir* region, whereas a modified T-DNA is included in another plasmid. The modified T-DNA contains the gene of interest and a marker gene between the T-DNA borders, which are necessary for release and transformation [21]. In 2004, this methodology was also used for gene disruption in *T. atroviride* [26] following a *Saccharomyces cerevisiae* protocol [20]. In 2007, a pBI-hph binary vector system containing the *hph* gene as marker and an ATMT protocol were established for the transformation of *T. reesei* [14]. In 2019, Wu et al. [27] described a new method using two different *A. tumefaciens* strains bearing two different vectors, combining two genetic manipulations in one step, saving 15–30 days.

When the ATMT and PEG-mediated transformation methods were compared in four different *Trichoderma* species [18], the stability of transformants and successful integration of DNA were dependent on the method and strongly influenced by the used selection marker. Further, there were substantial differences between the tested species, pointing out the genetic diversity of the *Trichoderma* genus and suggesting that transformation method selection should be established based on trials for different species and strains [18]. Colonies could be obtained for all four species (*T. harzianum*, *T. asperellum*, *T. atroviride*, and *T. longibrachiatum*) using ATMT or PEG-mediated transformation and hygromycin or phleomycin for selection. In general, hygromycin resistance led to more colonies that phleomycin, and *T. longibrachiatum* yielded the most transformants followed by *T. harzianum*, *T. atroviride*, and *T. asperellum*. A subsequent analysis of the phenotypical stability (retaining of resistance after 3 passages) revealed that up to 70% instable transformants had been obtained, depending on the species, transformation method and marker. A final Southern blot analysis demonstrated that the transformed markers were only integrated into the genome in some cases. In summary, *T. longibrachiatum* could be transformed with any method and any marker. Stable transformants for *T. harzianum* could only be obtained through PEG-mediated transformation, while the choice of marker influenced the stability of the *T. atroviride* and *T. asperellum* transformants stronger than the used transformation method [18].

### Biolistic transformation

During biolistic transformation or particle bombardment method, tungsten or gold particles are coated with DNA and injected into the cells at high velocity. It is an easy and convenient method, which circumvents the use of osmotically sensitive protoplasts, but the equipment and reagents are expensive. Therefore, it is usually considered only when other methods failed [12]. The first study on biolistic for filamentous fungi was done with *T. harzianum* and *Gliocladium virens* cells [16], and years later, it was also adapted to *T. longibrachiatum* and *T. reesei* [28]. This method increased transformation frequency and genetic stability compared with the protoplast-mediated transformation in *T. harzianum* [16]. The efficiency of this transformation method mainly lies on three parameters: the vacuum strength in the chamber, the distance travelled by the particles before hitting the cell and the size and density of the microparticle. In *T. reesei*, the best transformation results could be obtained with a distance of 3 cm, a helium pressure of 1350 psi and a DNA amount of 100 ng coated on 0.7 μm diameter tungsten particles, yielding 39 colonies per μg of circular DNA [29]. The authors used an adaptor that splits the helium shock wave over seven macrocarriers, uniformly spreading the particles over a larger area and, therefore, maximizing the number of cells transformed in one bombardment.

### Electroporation

Electroporation uses an electric shock to create micropores in the membrane, allowing the exogenous DNA to penetrate the cell. The correct choice of the field intensity is crucial for restoring the original membrane structure. Otherwise, the damage in the membrane will be irreversible, leading to cell death [12]. The electroporation protocol in filamentous fungi was established in *T. harzanium* cells [15]. Competent cells with partially digested cell wall are induced for DNA uptake with an electric pulse and addition of PEG, obtaining frequencies up to 400 transformants per μg of DNA [15]. Recently, another protocol for electroporation was developed. This protocol does not require the addition of PEG, making it less time consuming and, due to the re-usage of electroporation cuvettes, also less expensive [30].

## Selection markers

Marker genes allow the selection of successful transformation events. Commonly used selection markers in *Trichoderma* spp. are listed in Table 1. We differentiate between two types of markers in this review. Heterologous marker genes can either confer resistance against a selecting agent that prevents the growth of wild-type *Trichoderma* spp. or enable the utilization of an otherwise non-metabolizable nutrient source. Auxotrophic markers encode for enzymes that are part of a biosynthesis pathway of a primary metabolite (e.g., amino acids). Strains with a deficiency of these markers cannot grow without supplementation of corresponding metabolites. The complementation of the genes reestablishes prototrophy, and the fungus can grow on a minimal medium. While heterologous markers can be directly used in wild-type strains, auxotrophic markers work only in auxotrophic strains. On the downside, heterologous markers may change the physiology and metabolism of the transformed strains by adding enzymes that are not native to *Trichoderma*, and many selection agents are toxic and/or expensive. Once auxotrophic strains are obtained (by random mutations or targeted genetic modifications), the usage of auxotrophic markers is preferable, as they are less cost-demanding than heterologous markers, do not rely on the usage of potentially harmful substances, and do not change the physiology of the transformed cells.

**Table 1:** Commonly used selection markers in *Trichoderma* spp.

Gene	Selection principle	Type	Species	Refs.
*pyr4*	Uridine prototrophy, 5 ‘-FOA sensitivity	Aux	*Trichoderma reesei*	[59, 106]
*pyr2*	Uridine prototrophy, 5 ‘-FOA sensitivity	Aux	*Trichoderma reesei*	[4]
*asl1*	Arginine prototrophy	Aux	*Trichoderma reesei*	[59]
*ade2*	Adenine prototrophy	Aux	*Trichoderma reesei*	[4]
*his1*	Histidine prototrophy	Aux	*Trichoderma reesei*	[32]
*ben*	Benomyl resistance	Het	*Neurospora crassa*	[107]
*ble*	Bleomycin/phleomycin resistance	Het	*Streptoalloteichus hindustanus*	[108]
*hphB*	Hygromycin resistance	Het	*Escherichia coli*	[109]
*ptrA*	Pyrithiamine resistance	Het	*Aspergillus oryzae*	[110]
*nptII*	Geneticin/neomycin resistance	Het	*Escherichia coli*	[111]
*amdS*	Ability to use acetamide as N-source, fluoroacetamide sensitivity	Het	*Aspergillus nidulans*	[6]

**Fig. 3:**
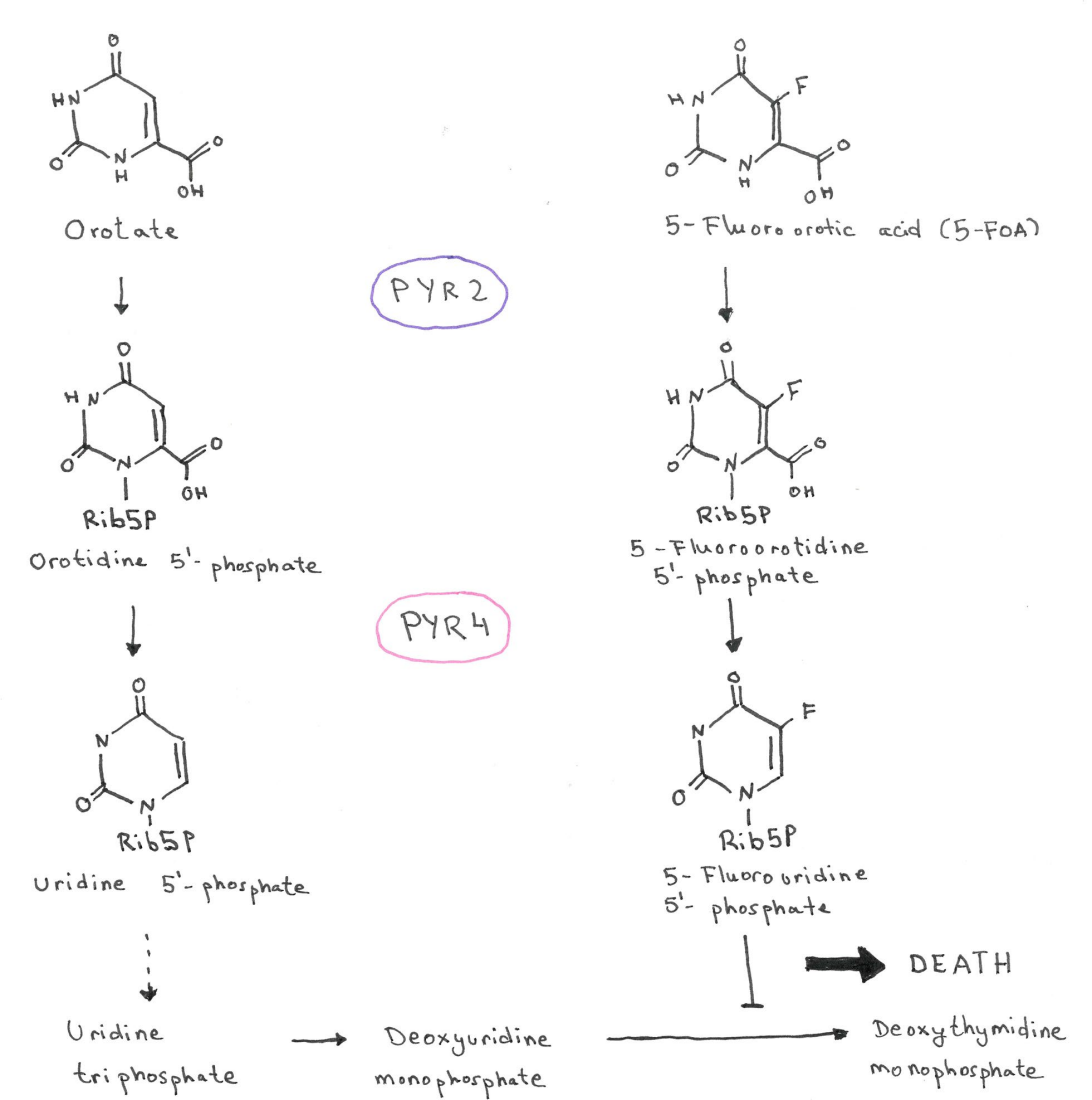
Pyrimidine biosynthetic pathway

Special cases are the bidirectionally selectable makers, *pyr4*, *pyr2*, and *amdS*. The auxotrophic markers *pyr4* and *pyr2* encode for enzymes involved in the pyrimidine biosynthesis, i.e., the orotidine 5'-phosphate decarboxylase and the orotate phosphoribosyltransferase, respectively (Fig. 3) [4]. They can be used for positive selection as described above, and additionally for negative selection (their absence) because the orotate phosphoribosyltransferase (encoded by pyr2) also accepts 5-fluoroorotic acid (5-FOA) as substrate resulting in the ultimate formation of the toxin 5-fluorouracil (Fig. 3). The orotidine 5'-phosphate decarboxylase (encoded by pyr4) catalyzes the second step of this pathway (Fig. 3). The heterologous marker *amdS* gene (from *Aspergillus nidulans*) enables *Trichoderma* spp. to use acetamide as nitrogen source but also makes it sensitive to fluoroacetamide [6].

## Marker recycling

Marker recycling allows the (re-)usage of a marker if multiple consecutive genetic manipulations are necessary by removing the marker gene after each transformation step. The markers *pyr4*, *pyr2*, and *amdS* [4, 6] have been used for that application due to the possibility to select for their presence and absence. There are three different approaches published in *T. reesei* with the aim of marker recycling and a fourth method, which was not explicitly designed for marker recycling but can still be used for it.

### Marker recycling using homologous recombination

**Fig. 4:**
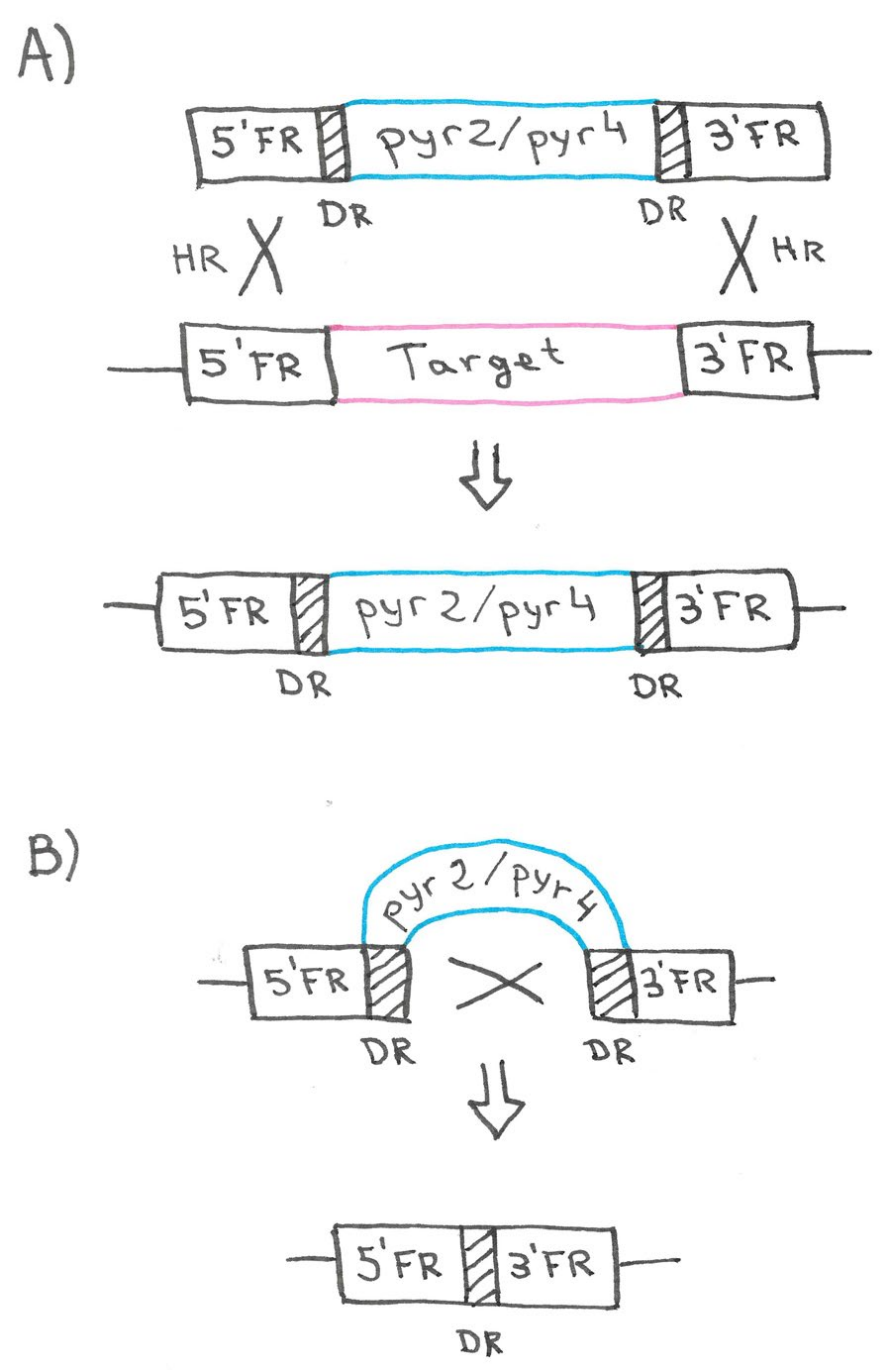
Gene deletion and marker recycling using a bidirectional marker. **A** The target gene is deleted using a homologous recombination (HR) strategy. A double cross-over must occur between the homologous sequences flanking the target gene (3′ and 5′ flanking regions, FR). The *pyr2* or *pyr4* gene replaces the target gene and restores uridine-prototrophy in an auxotrophic recipient strain. **B** For marker recycling, an internal cross-over between direct repeats (DR) flanking the marker gene needs to occur, resulting in 5-FOA resistance

The first system uses direct repeats of any sequence to facilitate the excision of a bidirectional marker (*pyr4* and *pyr2* in the published studies) [4, 31] via homologous recombination (Fig. 4). These systems work only in strains that are *pyr4* and *pyr2* deficient, respectively. The marker gene is flanked by a direct repeat of any sequence (a 380 bp fragment of the *Streptoalloteichus hindustanus* bleomycin gene in [31] or a 375 bp fragment of the *A. oryzae pyrG* promoter region in [4]). Notably, the used strains in these studies were non-homologous end-joining (NHEJ) deficient, enhancing the homologous recombination rate (we will discuss this aspect of strain constructions in the next section). A random cross-over may occur between the direct repeats, resulting in excision of the marker (Fig. 4B), for which can be selected with 5-FOA. A copy of the direct repeat remains in the locus (Fig. 4B). In a recent study, a short part of the 3’ flank was duplicated and added downstream of the 5’ flank. After successful gene deletion by a double crossover using the marker *pyrG*, the direct repeat was resolved by an internal homologous recombination leading to a seamless maker removal [32].

### Marker recycling using the Cre/loxP system

**Fig. 5:**
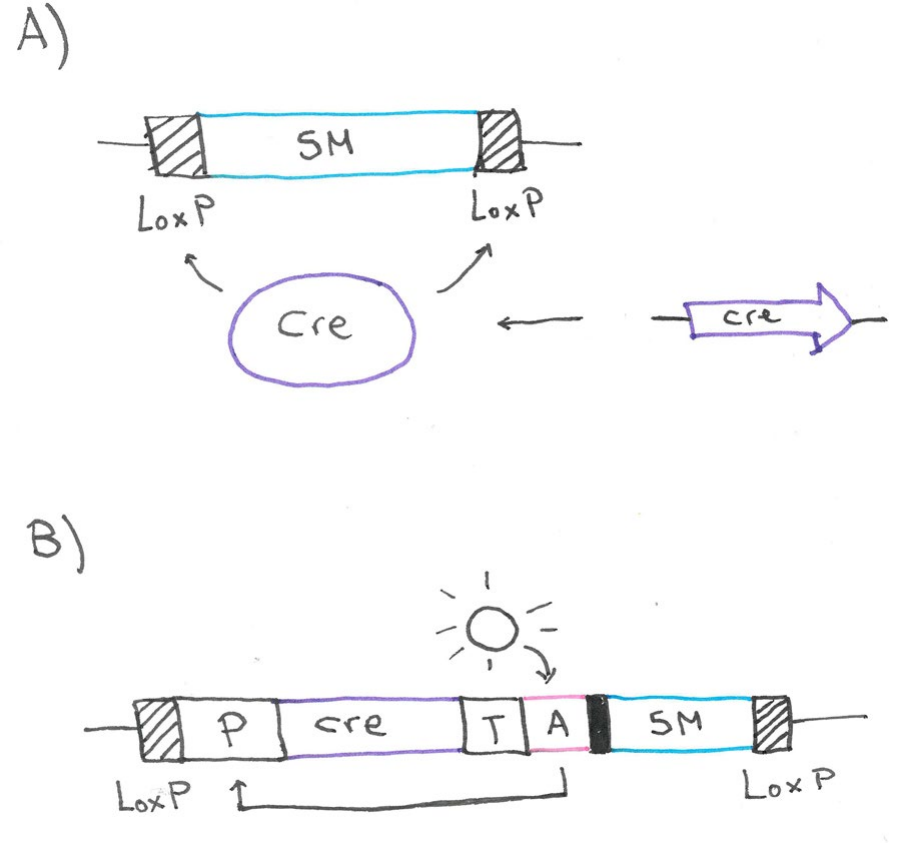
Marker recycling using the Cre/*loxP* system. **A** The selection marker (SM) is flanked by two direct repeats of a LoxP site. Recombination of the two LoxP sites by the Cre recombinase leads to excision of the selection marker. In this system, the gene encoding for the Cre recombinase is located in a different locus. **B** In the LML 3.0 system, the *cre* gene is part of the deletion cassette. A light inducible activator (A) induces expression of Cre, which leads to excision of the *cre* gene together with the SM. P, promoter; T, terminator

The second system utilizes the Cre/*loxP* system from the bacteriophage P1 [33] to enhance the excision rate of a bidirectional marker compared to the first system. The site-specific recombinase Cre recombines direct repeats of two LoxP sites flanking the marker (Fig. 5A) [34]. In the system developed for *T. reesei*, the recombinase gene was inserted into the genome, replacing the *pyr4* gene and put under the control of a xylan-inducible promoter. Consequently, cultivation on xylan induces the excision of the sequence between the LoxP sites, in this case, the *hph* and *amdS* marker [34]. Notably, a LoxP site remains in the locus. The main advantage of this system is the efficiency of the Cre used for marker excision, which is also a disadvantage. The number of LoxP sites grows with each transformation step and the risk of recombination between the altered loci. As the last step of the strain design, the *cre* gene can and shall be removed by retransformation with *pyr4* gene. The removal of *cre* is necessary to avoid unwanted recombination events in the fungus.

The LML3.0 system, an improvement of the Cre/*loxP* system, was published by Zhang et al. in 2016 [35]. They used mutation carrying LoxP sites that were no longer a substrate for Cre after the initial recombination, minimizing the risk of unwanted recombination events. Further, the *cre* gene was put under a light-inducible promoter and integrated into the excised sequence, thus eliminating the necessity for an ultimate removal of the *cre* gene from the genome. The high efficiency of the modified Cre/*loxP* system enables them to use only the unidirectional marker *hph* in the self-excising cassette without the need for counterselection (Fig. 5B).

### Marker recycling using the I-SceI meganuclease

This method is based on the usage of the *Saccharomyces cerevisiae* I-*Sce*I meganuclease. This enzyme is part of a homing intron occurring in the mitochondria of *S. cerevisiae* and cuts the 18-bp long recognition site 5'-TAGGGATAACAGGGTAAT-3’ [36]. Ouedraogo et al. [36] inserted *pyrG* flanked by two I-*Sce*I sites into *T. reesei* and could successfully remove *pyrG* via transient expression of I-*Sce*I from a non-integrated suicide plasmid.

## Extrachromosomal replication, ectopic and homologous integration

During the transformation of filamentous fungi like *Trichoderma* spp., the exogenic DNA can undergo different fates. The most probable fate is a non-integration event. The DNA remains extrachromosomal and is degraded by DNases and/or not replicated by the DNA-polymerase and thus lost. Notably, it is possible to drive gene expression for a short time from a suicide plasmid [36].

### Extrachromosomal replication using the AMA1 pseudo-ori

There is only a single exception to this loss of extrachromosomal DNA. The AMA1 sequence functions as a pseudo-ori in a broad range of filamentous fungi [37]. This sequence was isolated from a genomic library of *A. nidulans*, which enhances the transformation frequency up to 2000-fold compared with conventional integrating plasmids. AMA1-bearing plasmids were detected as circular molecules in the nuclei, are structurally stable, do not integrate into the chromosome and allow autonomous plasmid replication [38, 39].

### Ectopic integration

Transformed DNA can also be integrated randomly at any locus (ectopic integration). This ectopic integration is mediated by the NHEJ pathway, the primary DNA-repair mechanism in *Trichoderma* during the vegetative growth phase [40]. Consequently, after a transformation and selection procedure, most transformants carry at least one ectopically integrated copy of the transformed DNA in their chromosomes. Notably, there is a high chance of multiple integrations. These ectopic integrations can happen at undesired sites, i.e., the heterochromatin or coding or regulatory regions. For instance, genes integrated into heterochromatin will not be transcribed. In contrast, the integration at already functional sites can lead to unwanted genetic manipulations (loss-of-function mutations by disruptions or gain-of-function mutations by direct or indirect deregulation of gene expression).

Interestingly, the ectopic integration allows co-transformation of two plasmids, one containing the marker and the other bearing the interest gene [6]. Co-transformation rates of up 80% have been reported in *T. reesei* [6]. Due to the randomness of the (co)-integration, resulting transformants need to be carefully screened and tested, and it is vital to use several different transformants for subsequent experiments. A strategy that allows a semi-targeted insertion is the introduction of DNA double-strand breaks via restriction endonucleases. To this end, the exogenic DNA is digested with a restriction enzyme prior to the transformation, and the same enzyme is added to the transformation reaction. The enzymes can be taken up and cut the DNA in vivo. The exogenic DNA and the digested chromosomal DNA have matching ends, which leads to integration at any target site(s) of the restriction enzyme [41].

### Homologous integration

The most unlikely fate of transformed DNA is targeted integration via homologous recombination. To this end, the DNA sequence to be inserted must be flanked by DNA sequences homologous to the target site. This method must be used for targeted deletions, disruptions, or replacements and can be used for targeted integration of expression cassettes. Homologous recombination commonly occurs in filamentous fungi during meiosis and the final stage of mitosis (S-Phase) [42]. However, meiosis is not occurring during transformation and the S-phase only in a small proportion of protoplasts. Consequently, homologous recombination and thus targeted integration is a rare event during the transformation of *Trichoderma* spp. There are four strategies suggested and used to enhance the chance of homologous recombination in *Trichoderma*.

#### Split marker

The first strategy is the usage of split markers. To this end, the transformation cassette is split into two parts. The first part consists of the 5’flank of the target site and approximately two-thirds of the marker. The second part consists of two-thirds of the marker and the 3’flank. The marker parts on both constructs have an overlapping region (middle third). Each part is non-functional itself, but after homologous recombination at the middle third, the full-length marker is assembled. In order words, the marker will only function in cells that are in a cell cycle phase during which homologous recombination is active. Consequently, the chances of integration of the integration cassette are statistically higher.

#### NHEJ-deficiency

The second strategy to enhance homologous recombination is to delete a gene of the NHEJ pathway. In *Trichoderma* spp., strains bearing deletions of either *ku70*, *ku80* or *mus53/Lig4* [34, 40, 43, 44] are available. In all three cases, high rates of homologous recombination (up to 100%) were reported. However, these strains have a higher UV sensitivity, as they lack an important DNA repair mechanism, affecting fundamental cellular aspects such as telomere maintenance, nuclear spatial organization and mitotic recombination [45, 46]. To solve this problem, Chum et al. developed a transient silencing method to knockdown the mRNA levels of the latter mentioned DNA repair genes instead of permanent gene deletions. The authors transfected protoplasts, mycelium and even spores with small interfering RNAs and could obtain relatively high levels of homologous recombination [47]. An alternative strategy was developed by Zhang et al. together with the previously mentioned LML 3.0 [35]. The so-called “OFN1.0” allows turning on and off the NHEJ pathway by reversible inversion of the *ku70* gene, mediated by two inverted LoxP sites, flanking the open reading frame. During the transformation steps, the *ku70* is in the OFF position (inverted). Upon self-excision of Cre, the *ku70* gene is reversibly inverted; 50% of the resulting final transformants carry the *ku70* in the correct orientation and are not NHEJ-deficient anymore. Notably, the flanking LoxP sites cause a reduction of the *ku70* transcription rate [35].

#### Introduction of double-strand breaks

The third strategy relies on the previously mentioned *S. cerevisiae* I-*Sce*I meganuclease. In a first transformation step, the I-*Sce*I recognition site is inserted at the target site. Notably, this transformation step is relying on the native, low recombination rate of *Trichoderma*. During the second transformation step, a high homologous recombination rate at the inserted I-*Sce*I recognition site can be achieved by transient expression of I-*Sce*I from an unstable suicide plasmid [36]. The obvious limitation of this method is a single, predetermined target site, which can only be used for integrations.

The fourth strategy to enhance the chance of homologous recombination is the CRISPR/Cas9 system, which introduces a double-strand break at the target site. This works analogously to the I-*Sce*I-mediated introduction of double-strand breaks with the advantage of the flexibility of CRISPR/Cas9. We discuss this in the following chapter.

## Application of the CRISPR/Cas9 system for genome editing

The CRISPR (clustered regularly interspaced short palindromic repeats) system is an adaptative immune system in bacteria and archaea. In 2012, the CRISPR system from *Streptococcus pyogenes* was suggested to be useable for programable genome editing [48]. The *S. pyogenes* CRISPR system consists of the endonuclease Cas9 (CRISPR-associated gene 9) and two interacting RNAs. The two RNA structure directs Cas9 to its target site, where it introduces a double-strand break. Notably, a single guide RNA (sgRNA) can be constructed and used to direct Cas9. The target site is a 20-bp sequence directly upstream of a protospacer-adjacent motif (PAM), in the case of *S. pyogenes* Cas9, 5’-NGG-3’. Since then, many studies have demonstrated the suitability and efficiency of genome-editing using CRISPR/Cas9 systems in several organisms, including various filamentous fungi [49–54].

**Fig. 6:**
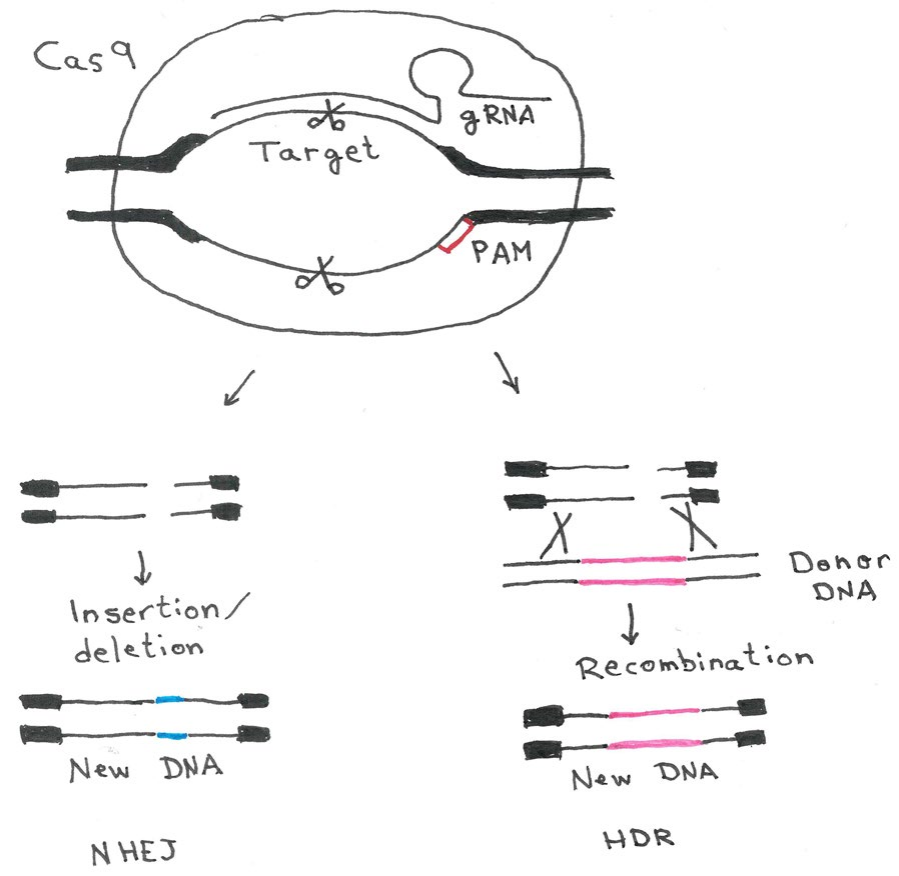
CRISPR/Cas9-mediated genome editing. The Cas9 endonuclease is programmed by the guide RNA (gRNA) to introduce a double strand (DSB) in a 20 bp long target site, directly adjacent to a protospacer adjacent motif (PAM). The DSB can be repaired by the error-prone non-homologous end joining (NHEJ) or via homologous direct repair (HDR), if a suitable donor DNA is present

There are two principal possible strategies for CRISPR/Cas9 genome editing (Fig. 6) [48]. For the first strategy, Cas9 is used to cut the target site, which is repaired by the NHEJ repair mechanism, and again cut by Cas9 and so on, until a mutation is introduced. These mutations usually are deletions that can lead to loss of function of the target gene (either by frameshifts or the deletion of essential amino acids in the respective protein). In the second strategy, CRISPR/Cas9 is used to introduce a double-strand break in the target site, analogously to the previously discussed I-SceI mediated strategy to enhance the recombination rate at the target site. Here, a repair matrix is offered, leading to the deletion or modification of the target gene.

For *T. reesei*, different approaches and strategies to apply CRISPR/Cas9 have been suggested. In 2015, a codon-optimized gene encoding for Cas9 under the control of an inducible promoter was introduced into the genome of *T. reesei* QM6a and Rut-C30. The sgRNA was transcribed in vitro and transformed into Cas9-expressing strains using a modified PEG-mediated transformation [55]. In this study, both before mentioned strategies were demonstrated to work in NHEJ-positive *T. reesei* strains. The authors reported homologous recombination rates above 90% when using flanks with lengths as short as 200 bp [55]. In following studies, three groups published methods for genome modifications in *T. reesei* using in vitro assembled Cas9/sgRNA complex, also called Cas9 ribonucleoprotein, intending to minimize the risk of off-target mutations [49, 50, 53]. Zou et al. demonstrated that very short flanks (20 bp) could be used for gene deletions in combination with the Cas9 ribonucleoprotein [53]. Two other groups suggested using RNA polymerase III promoters for in vivo transcription of the sgRNA [56, 57], in order to circumvent possible problems with in vitro transcribed sgRNA uptake and stability. In these studies, a plasmid containing an sgRNA expression cassette under the control of an RNA Pol III promoter was either transformed into an Cas9-expressing strain [56] or co-transformed together, with another plasmid bearing the cas9 gene [57]. Regardless of the used method, the CRISPR/Cas9 system promises to enhance the genome editing capabilities in *Trichoderma* spp.

The CRISPR/Cas9 system of course also has some limitations (e.g., the consensus sequence of the PAM site is 5’-NGG-3’, which makes it hard to find suitable sgRNA target sites in AT-rich regions, or the chance of off-target cuts). Alternative nucleases, such as Cas12 and Cas13 might solve these problems. For instance, Cas12 offers the possibility of targeting T-rich PAM sequences [58]. Notably, these alternative nucleases have not yet been used in *Trichoderma* spp., but they will undeniably play an important role in genome editing of these fungi in the close future.

## Targeted insertions using auxotrophic markers

**Fig. 7:**
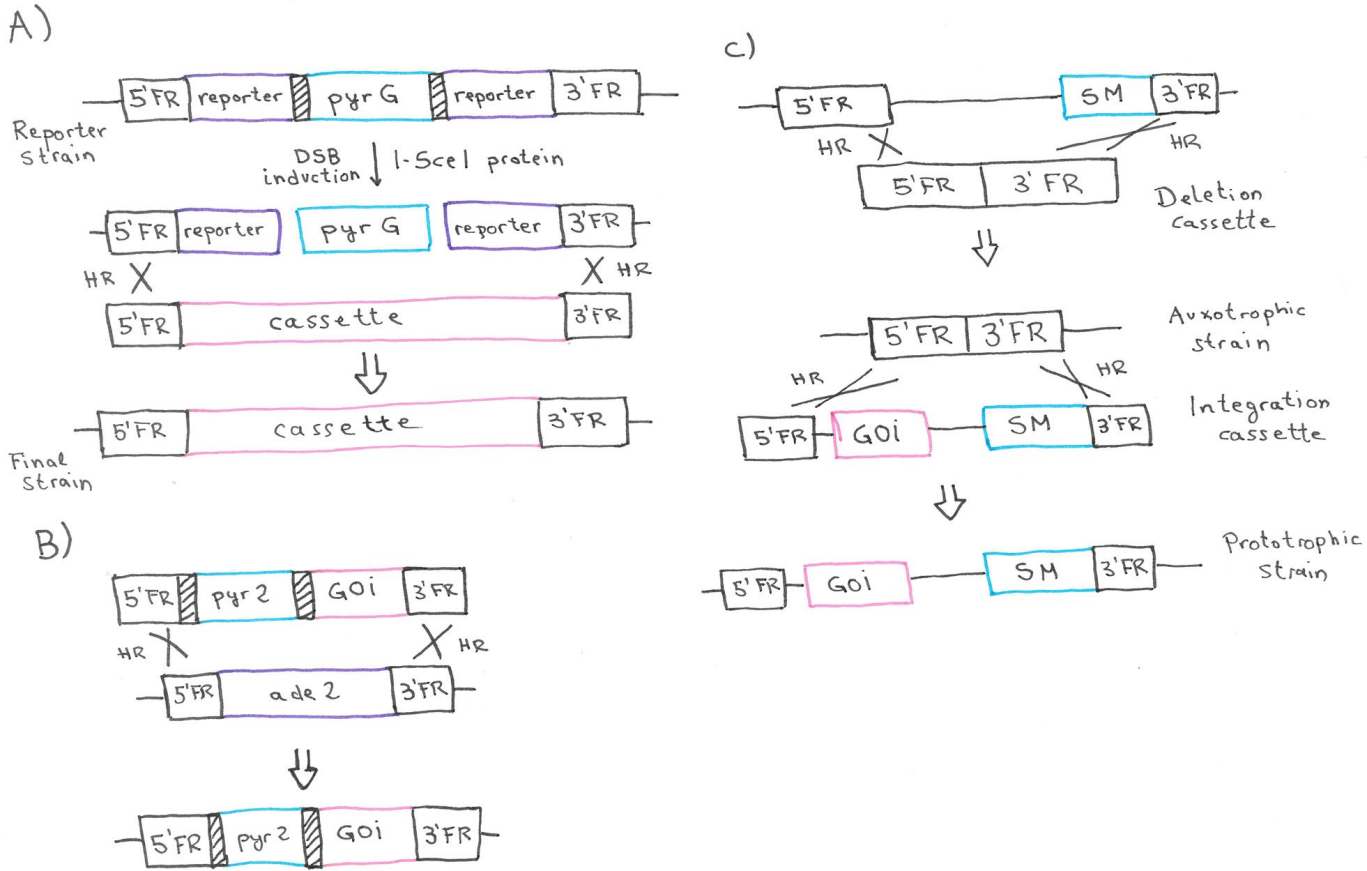
Strategies for targeted gene insertions. **A** The reporter strain carries the *pyrG* gene flanked by two I-SceI recognition sites (shaded boxes) and two halves of a reporter genes. Transient expression of the I-SceI leads to doubles strand breaks (DSB). An expression cassette can be integrated via homologous recombination (HR) using homologous flanking regions (3’ and 5’ FR). **B** The gene of interest (GOI) is inserted together with the *pyr2* marker into the *ade2* locus via HR, resulting in a gene deletion. In this system, the *pyr2* gene is flanked by direct repeats that may be used for maker recycling. **C** For the insertion of a GOI into the locus of an auxotrophic selection marker (SM), first the SM gene needs to be deleted. Then, an integration cassette containing the GOI and the previously deleted SM, are integrated into genome of the auxotrophic strain, reestablishing prototrophy

As mentioned above, a commonly used strategy for gene insertions is the co-transformation of two plasmids, one containing the marker and the other one bearing the gene of interest. This results in most of the cases in random integration and leads to variable copy number and integration sites, which translates into variation production levels [6]. Moreover, the integration of the marker does not assure integration of the gene of interest. This results in subsequent laborious work for the analysis and seek of a positively transformed strain. An obvious way to solve and circumvent these problems is targeted gene integrations. Ouedraogo et al. developed the I-*Sce*I-mediated integration system for this purpose (Fig. 7A) [36]. An alternative is the utilization of auxotrophic markers, which can be used simultaneously as integration sites and for selection. This method demands high rates of homologous recombination and has only been described in NHEJ-deficient strains in *Trichoderma* spp. [36]. We speculate that these strategies and methods could also be performed in NHEJ-positive strains if CRISPR/Cas9 is used to open the target sites. Jørgensen et al. described the possibility to integrate an expression cassette at the *ade2* locus, using *pyr2* as marker (Fig. 7B). Notably, this method yields adenine-auxotrophic strains [4]. Derntl et al. [59] developed a similar system, which allows integration of expression cassettes upstream of the *pyr4* and *asl1* genes. Therein, the markers for the integration are the previously deleted auxotrophic markers. The resulting strains regained prototrophy (Fig. 7C).

## Promoters used for heterologous gene expression

### Native promoters

An obvious necessity is the availability of suitable promoters. Traditionally, promoters are classified into constitutive and inducible. Constitutive promoters are always expressed independently of environmental factors, whereas inducible promoters respond to external biotic or abiotic factors. Depending on the aim of the gene expression, constitutive or inducible promoters might be favorable.

*In T. reesei,* different constitutive promoters, with different strength are commonly used, for instance the *eno1* (enolase) [60], *gpd1* (glyceraldehyde-3-phosphate dehydrogenase) [60], *tef1* (transcription elongation factor 1α) [61], *pki1* (pyruvate kinase) [62] and *pdc1* (pyruvate decarboxylase) [60] promoters. The strength of *eno1*, *gpd1* and *pdc1* promoters was assessed in *T. reesei* via expression of *xyn2* under the control of these promoters. The highest xylanase activity was achieved when *xyn2* was under the control of the *pdc1* promoter (9266 IU/ml). Using *eno1* promoter, *T.reesei* produced 8866 IU/ml of xylanase activity, and the lowest value was detected when using *gpd1* promoter (686 IU/ml) [60].

Inducible promoters offer a wider variety of choice. As *T. reesei* is a well-known cellulases and xylanases producer, several inducible promoters originate from genes encoding for these proteins. The strength of cellulase cellobiohydrolase *cbh1* (*cel7a* according to the CAZyme annotation [63]) promoter makes it the inducible promoter of choice [64]. Multiple copies can be introduced, but more than four copies will saturate the system due to the deletion of transcriptional activators [65, 66]. This promoter can be induced by different carbon sources such as cellulose [67], lactose [68] and sophorose [69] and shut-off by glucose in a Cre1-positive background [70]. There are a few other frequently used cellulase promoters, including P*cel6a* or P*egl2*. Besides their lower expression strength, all of them have similar advantages and limitations as the promoter of *cel7a* [71].

There are also several xylanase promoters in use, e.g., P*xyn1*, P*xyn2* and *Pxyn3* [71]. *Pxyn1* and *Pxyn3* are repressed by CRE1 [72], while XYN2 retains a low constitutive expression on D-glucose, and it is induced by cellulose [73]. While D-glucose represses these promoters, the *Pstp1* is induced by it. Stp1 is a sugar transporter, and studies on it are of interest, as it allows the use of cheap D-glucose as activating agent [71, 74].

A native promoter outside of the cellulase and hemicellulose system is the promoter of the *tcu1* gene encoding for a putative copper transporter [75]. The system is highly sensitive to copper levels in the media and can regulate the expression of heterologous and homologous proteins.

### Engineered promoters

Native promoters have their given properties and limitations; by modifying and engineering these native promoters, additional favorable properties can be added or unfavorable properties removed. A common approach to modify natural promoters is the deletion of repression mechanisms. For instance, the strength of the *xyn1* and *cbh1* promoter can be enhanced by the deletion of the CRE1 binding sequence [76]. In 2018, Kiesenhofer et al. tested the influence of some cis-elements in the *cbh1* promoter on its strength and inducibility [77]. By inserting a cis site from the *xyn1* promoter, the engineered promoter could be induced by xylan and reach higher expression levels than the native *cbh1* promoter.

Another example of an engineered promoter is the already described LML3.0 light-inducible system [35]. In this system, *cre* is transiently expressed for marker recycling, as discussed before. In 2014, Wang et al. [78] described a blue light-mediated regulation of DNA transcription in *T. reesei.* This system is composed of the DNA-binding domain of Gal4, light oxygen-voltage (LOV) domain of Vivid (which regulates blue-light responses in *Neurospora crassa*), a linker, and the VP16 activation domain. This synthetic protein (G1V) was expressed under the control of *pki* promoter and the *nos* terminator was used. This system was later implemented in the already described LML3.0 system for recycling markers [35].

### Synthetic expression systems

The utilization of native and engineered promoters to drive heterologous gene expression is very convenient and has high success rates, but there are two problems. First, the introduction of several copies of a promoter is limited due to the depletion of transcriptional activators and the risk of intra- and inter-chromosomal recombination, especially in an NHEJ-deficient strain. Second, using a native expression system hinders the study of this native expression system and involved regulatory factors. Consequently, synthetic expression systems have been developed and used in many organisms [79–81]. An ideal synthetic expression system does not contain any native elements that could lead to interference, does not rely on or react to native stimuli, and can be reversibly and gradually controlled with no basal expression levels and high maximum expression levels. A widely used system in eukaryotes is the Tet-on/off system [82]. This system consists of a synthetic transcription factor that reacts to tetracycline and its derivate doxycycline and a synthetic promoter containing the corresponding binding sites. This system has been modified and optimized for the application in *A. fumigatus* [83] and *A. niger* [84], and was also successfully used in other fungi [85]. However, it has not been used in any *Trichoderma* species to date.

In 2018, Rantasalo et al. [86] developed a universal expression system for yeast and filamentous fungi (*T. reesei* and *A. niger*). In this synthetic expression system, a synthetic transcription factor is expressed constitutively, while the gene of interest is put under the control of a synthetic promoter. The synthetic transcription factor consists of the DNA-binding domain of BM3R1, a nuclear localization signal, and the transactivation domain of the Herpes simplex virus protein VP16. Different synthetic promoters containing the BM3R1 binding sites with different strengths are available [86]. While this system was demonstrated to reach very high expression levels, it lacks a mechanism to control and induce gene expression.

## Polycistronic expression

**Fig. 8:**
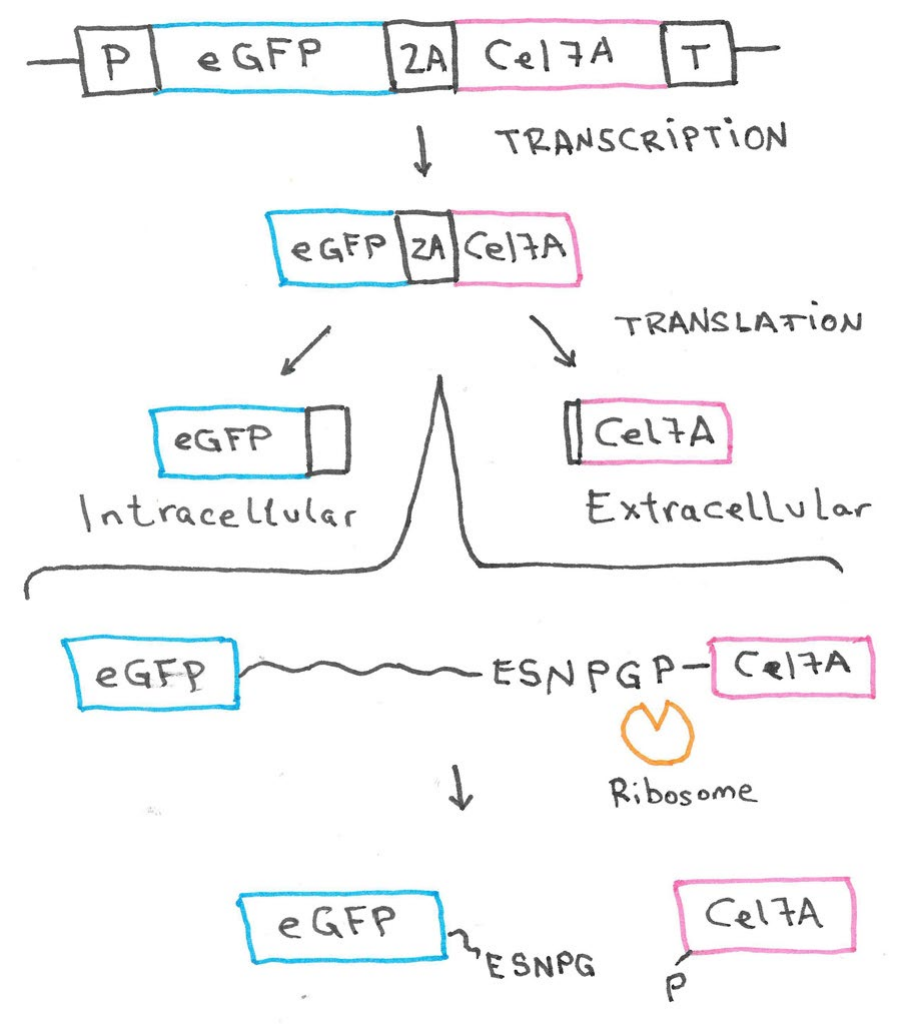
Usage of a 2A peptide in a bicistronic gene construct yielding two independent proteins. The coding regions for eGFP and the cellulase Cel7a are transcribed together using the same promoter (P) and terminator (T). The coding regions are spanned by the genetic sequence for a 2A peptide. During the translation, the 2A peptide leads to a “skipping” of the ribosome; it fails to make a peptide bond between the C-terminal proline and the previous amino acid in the 2A peptide

When a compound shall be heterologously produced or a metabolic pathway redirected or engineered, typically, several genes of interest must be expressed simultaneously. Given that eukaryotes use monocistronic mRNAs, each of these genes must be expressed from an individual promoter and equipped with an individual terminator. This makes the construction of the expression cassettes and the transgenic strains highly laborious and time-consuming. In prokaryotes, operons and polycistronic mRNAs allow the expression of several genes from a single promoter. In *T. reesei* and other filamentous fungi, a pseudo-polycistronic expression system can be used. To this end, the 2A peptides from the foot-mouth disease virus (FMDV) and other picornaviruses are introduced in frame between two or more coding regions, without any stop codons in between. They can manipulate the ribosome, preventing the formation of the glycyl-prolyl peptide bond at the C-terminal part of the 2A peptide. This results in the release of the nascent protein, which is tagged at the C-terminus with the greatest part of the 2A peptide [87]. The following protein has a proline attached to the N-terminus (Fig. 8). The 2A peptide from FMDV is 18 amino acids long (LLNFDLLKLAGDVESNPG) [88], but there are several different sequences available and used, as the presence of the motif (-DxExNPGP-) is enough to cause the cleavage. The most widely used 2A sequences are the ones derived from FMDV (F2A), thosea asigna virus (T2A), equine rhinitis virus (E2A) and porcine teschovirus-1 (P2A) [89]. 2A peptides were successfully implemented in *T. reesei* in 2017 [90]. In the study, two proteins (CBHI and eGFP) were expressed in *T. reesei* separated by 2A peptides with a cleavage efficiency of almost 100%. CBHI was correctly secreted and functional while eGFP remained intracellular, being able to screen for transformants in one single step analysis [90]. The approach can be extended for the expression of multiple proteins by adding more 2A peptide sequences between several genes, as successfully used in *A. nidulans* [91].

## Codon optimization

The use of codons varies between different organisms. This issue has to be considered for efficient heterologous expression of proteins. *Trichoderma* genes show a bias against adenine or thymine at the codon wobble position. This may negatively affect the expression of genes with high AT content in *Trichoderma* [92]. Formation of truncated mRNA transcripts has been reported when expressing genes with a high content of A and T in yeast hosts as well as in *A. awamori* [93, 94]. However, the gene sequence can be modified to include G or C at the third codon position without altering the amino acid sequence of the protein. This approach has been followed in *Trichoderma* species to express genes from other organisms. For instance, for the heterologous expression of the *xynB* gene from *Dictyoglomus thermophilus* in *T. reesei,* the AT content was lowered from 61 to 40% [92]. In another work, the native and a codon-optimized version of the mannanase genes (*AnMan5A*) from *A. niger* were expressed and compared in *T. reesei*. Notably, the codon optimization enhanced expression and activity threefold [95]. These works clearly show that codon optimization is an essential factor determining the success of the expression and activity of heterologous proteins expressed in *T. reesei*. However, the non-optimal codon usage in the native host may be important for correct folding and activity and should be considered. Zhou et al. found that codon-optimization of the *frq* gene, encoding for the FREQUENCY (FRQ) clock protein in *Neurospora crassa*, resulted in enhanced expression but impaired FRQ function, which was explained by misfolding of the protein [96].

## Terminators and 3’untranslated regions

Terminators and 3’untranslated regions in eukaryotes are not only necessary for a successful termination of transcription and as docking sites for the poly-A-tail but also control mRNA stability and can influence where the mRNA is translocated [97]. Despite the biological importance of these elements, there is not much information and data available on the influence of the terminators on gene expression in *Trichoderma*. Commonly used terminators originate from the two natively highly expressed genes, *cbh1* and *cbh2* [98–100], but other terminators were also used for heterologous protein expression, such as *goxA* terminator [59] and *nos* terminator [35].

## Protein secretion

**Fig. 9:**
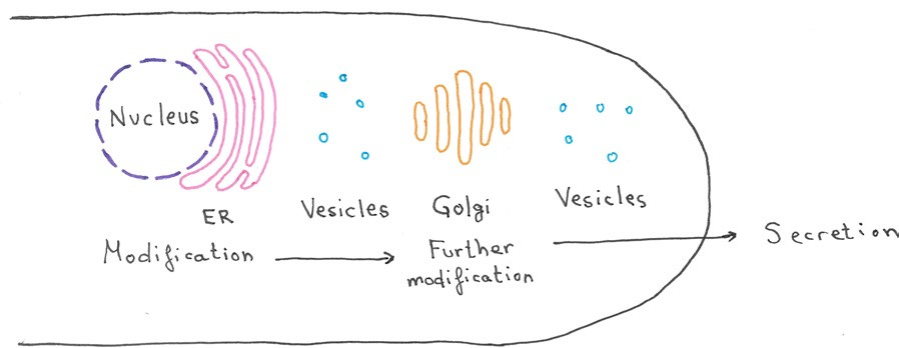
Schematic view of the protein secretion route in *T. reesei*. A nascent protein is translated into the endoplasmic reticulum (ER) and transported via vesicles to the Golgi apparatus and further to the tip of a hyphae where it is secreted via exocytosis

Filamentous fungi generally have high protein secretion capabilities as a consequence of their osmotrophic lifestyle [101]. As mentioned before, *T. reesei* holds the record for the highest protein production (and therefore secretions) rates, with up to 100 g/l in industrial bioprocesses [102]. In fungi, the protein secretion of the strongest expressed extracellular protein starts with a signal sequence at the N-terminus of nascent proteins. This signal peptide is recognized by the signal recognition particle, which triggers co-translational translocation. The ribosome-mRNA-peptide complex is translocated to the endoplasmic reticulum (ER), resulting in a nascent polypeptide chain translocated into the ER lumen. In the ER, the proteins are correctly folded, and some potential post-translation modifications are added. In the following steps, the extracellular proteins are trafficked through the Golgi apparatus to the cell membrane via vesicular transport (Fig. 9). A comprehensive review about the protein secretion in *T. reesei* can be found in [102].

Taking advantage of the excellent natural secretion capacity of cellulases and xylanases in *T. reesei*, some early studies fused the CBHI cellulase promoter and other domains to different proteins to increase their secretion rate. In 1995 [103], short and heavy Fd chains of Fab molecules were fused to the C-terminus of CBHI core-linker region, obtaining a 50-fold increase in the secretion of these molecules. The fusion might increase the steady-state levels of mRNA as well, and the linker region ensures separation and proper folding of the product from the CBHI core. The overall positive effect of the fusions could be explained by more efficient transcription, ER entry or folding, or passage through the secretory pathway in general [103].

Efforts for enhancing the protein production in *T. reesei* have focused on the transcription, but only to some extent on the secretion pathway. In 2017, a study tested the influence of different key proteins and factors for protein secretion on the heterologous protein production in *T. reesei* [104]. The *A. niger* glucose oxidase (GoxA) was heterologously expressed in *T. reesei,* and *snc1*, *bip1,* and *hac1* were overexpressed. SCN-1 is a v-SNARE, playing an important role in vesicle fusion from the Spitzenkörper to the plasma membrane, one of the last steps in the secretion pathway [102]. Overexpression of SCN-1 resulted in improvement of the oxidase expression. The same result was also observed in *S. cerevisiae* [105]. However, the overexpression of SCN-1 leads to a decreased cellulase and glucanase activity, suggesting a differential role in the secretion of native and heterologous proteins. BIP1 is a chaperone from the ER, and HAC1 is a transcription factor regulating unfolded protein response. Overexpression of these proteins also enhance the secretion of GoxA, but not as strongly as SCN-1 overexpression. In this work, the regulation of one secretion factor affects the expression of other elements, highlighting the complex regulation of the whole secretory pathway.

## Conclusions

In recent years, the progress in molecular genetics and synthetic biology has allowed the development of several tools to facilitate the engineering of genes and pathways and the heterologous expression of proteins. The CRISPR/Cas revolution has drastically changed the game rules, facilitating the genome editing of filamentous fungi. Synthetic promoters and transcription factors enable a controllable and robust expression of heterologous proteins, and the usage of auxotrophic selection markers allows targeted integration and the performance of multiple rounds of transformations in the same strain. However, some aspects of heterologous protein production, such as secretion, are still understudied and should be investigated in more detail in the future.

## Data Availability

Not applicable.

## Abbreviations

5-FOA: 5-Fluoroorotic acid
ATMT: *Agrobacterium tumefaciens*-Mediated transformation
Cas9: CRISPR-associated gene 9
CRISPR: Clustered regularly interspaced short palindromic repeats
GRAS: Generally recognized as safe
LOV: Light oxygen-voltage
ER: Endoplasmic reticulum
FMDV: Foot-mouth disease virus
NHEJ: Non-homologous end-joining
PAM: Protospacer-adjacent motif
PEG: Polyethylene glycol
sgRNA: Single guide RNA
